# High-resolution visualisation of antisense oligonucleotide release from polymers in cells†

**DOI:** 10.1039/d3sc06773d

**Published:** 2024-08-28

**Authors:** Jessica J. King, Kai Chen, Cameron W. Evans, Marck Norret, Ruba Almasri, Nathan J. Pavlos, Henry YL. Hui, Qiongxiang Lin, Uditi Bhatt, Stephen G. Young, Nicole M. Smith, Mehran Nikan, Clive A. Prestidge, Haibo Jiang, K. Swaminathan Iyer

**Affiliations:** a School of Molecular Sciences, The University of Western Australia Perth WA 6009 Australia swaminatha.iyer@uwa.edu.au; b ARC Training Centre for Next-Generation Biomedical Analysis, The University of Western Australia Perth WA 6009 Australia; c UniSA Clinical & Health Sciences, University of South Australia Adelaide SA Australia; d School of Biomedical Sciences, The University of Western Australia Perth WA 6009 Australia; e Translational Cancer Pathology Laboratory, School of Biomedical Sciences, The University of Western Australia Perth WA 6009 Australia; f Department of Medicine, University of California Los Angeles CA 90095 USA; g Ionis Pharmaceuticals, Inc. Carlsbad CA 92010 USA; h Department of Chemistry, Faculty of Science, University of Hong Kong Pok Fu Lam Hong Kong hbjiang@hku.hk

## Abstract

Antisense oligonucleotides (ASOs) are a well-established therapeutic modality based on RNA interference, but low cellular uptake, limited ability to direct ASO trafficking, and a range of intracellular barriers to successful activity compromise both gene silencing outcomes and clinical translations. Herein, we demonstrate that polymers can increase ASO internalisation *via* intracellular trafficking pathways that are distinct from lipid-based delivery reagents. For the first time, we spatially define internalisation and dissociation stages in the polymer-mediated cytosolic delivery of ASOs using Nanoscale Secondary Ion Mass Spectrometry (NanoSIMS), which enables visualisation of ASO localisation at the organelle level. We find that polymer–ASO complexes are imported into cells, from which free ASO enters the cytosol following complex dissociation. This information enables a better understanding of the intracellular trafficking pathways of nucleic acid therapeutics and may be exploited for therapeutic delivery to enhance the effectiveness of nucleic acid therapeutics in the future.

## Abstract 2

We explore polymer-mediated cytosolic delivery of antisense oligonucleotides using NanoSIMS, visualising the internalisation and dissociation of nucleic acid–polymer complexes at the subcellular level.

## Graphical abstract

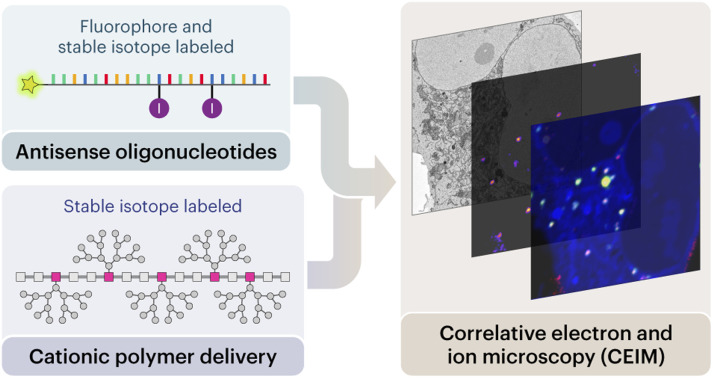

## Introduction

Targeted gene knockdown using nucleic acid therapeutics has been successfully demonstrated in cellular, animal, and human trials, showing promise for the regulation of therapeutic targets, including targets that cannot be modulated by small molecules. For example, antisense oligonucleotides (ASOs) can exert RNA interference effects by blocking the translation of RNA, producing inactive splice variants, or instigating the degradation of target RNA sequences, thereby silencing the expression of either protein or noncoding RNAs.^1,2^ ASOs are short, single stranded nucleic acid oligonucleotides (12–30 nt) designed to bind specifically to a target RNA molecule through base pairing.^3^ There are currently only nine clinically approved ASOs on the market, despite the discovery of the first gene knockdown by ASOs over 40 years ago. ASO efficacy is hindered by inefficient cellular uptake in most cell types and tissues;^4,5^ low cellular uptake results in insufficient ASO concentrations at the active site, compromising the effectiveness and durability of the therapeutic outcome.^6^

One barrier to the intracellular delivery of ASOs is the lipophilic nature of the cell membrane, which presents a hurdle for the transit of these anionic molecules. However, third generation ASOs, which are completely modified through phosphorothioate backbone linkages, help to overcome the barrier presented by the cell membrane to some extent.^5^ ASO internalisation *in vitro* in the absence of a delivery reagent occurs through a combination of fluid-phase (pinocytosis), caveolae potocytosis, adsorptive, and receptor-mediated endocytosis, with the specific uptake mechanism dependent on the chemical structure of the ASOs.^4,7^ However, some of these uptake pathways are nonproductive, as not all internalised ASOs are able to reach and interact with their intended targets.

The efficacy of ASOs is heavily influenced by their intracellular trafficking destinations.^8–10^ Nonviral delivery reagents based on cationic polymers can substantially improve the rate of ASO internalisation, and also help to target cargoes to specific intracellular trafficking pathways. Dendronised polymers have been established as promising gene delivery vehicles for DNA due to their flexibility in structural conformation and positive charge.^11^ Successful delivery is dependent on the ratio of polymer to ASO, expressed through an N/P ratio, *i.e.*, the number ratio of primary amines on the polymer to the number of phosphorothioates on the ASO backbone. However, while uptake can be improved using cationic polymers through enhanced cell membrane association, such an approach also targets cargoes towards endolysosomal pathways, many of which are unproductive. Once internalised, cargoes are trafficked into early endosomes where they face diverse outcomes. These include recycling back to the plasma membrane *via* recycling endosomes, trafficking to the trans-Golgi network, transport to late endosomes for lysosomal degradation, or delivery to multivesicular bodies (MVBs) for exocytosis.^7^ Thus, understanding trafficking and directing cargoes to particular pathways, whether the ASO is delivered natively or with the assistance of a delivery agent, are critical considerations in enhancing therapeutic effectiveness.

Ion microscopy (IM) using Nanoscale Secondary Ion Mass Spectrometry (NanoSIMS) enables the direct detection, visualisation, and quantification of stable isotope-labelled molecules with a lateral resolution of ∼40 nm.^12–15^ When combined with electron microscopy for visualisation of cell ultrastructure, correlative electron and ion microscopy (CEIM) imaging workflows allow for the simultaneous mapping of morphological and chemical information of biological samples with nanoscale precision.^16^ In this study, we use CEIM to spatially define the accumulations of ASOs across subcellular compartments, to establish delivery mechanisms and attempt to understand ASO trafficking in the context of polymer-assisted delivery. Using duplexed labelling of both the delivery agent (^79^Br-labelled polymer) and cargo (^127^I-labelled ASO), we, for the first time, enable visualisation of the ASO intracellular delivery and subsequent cytosolic release to facilitate therapeutic activity.

## Results and discussion

**Fig. 1:**
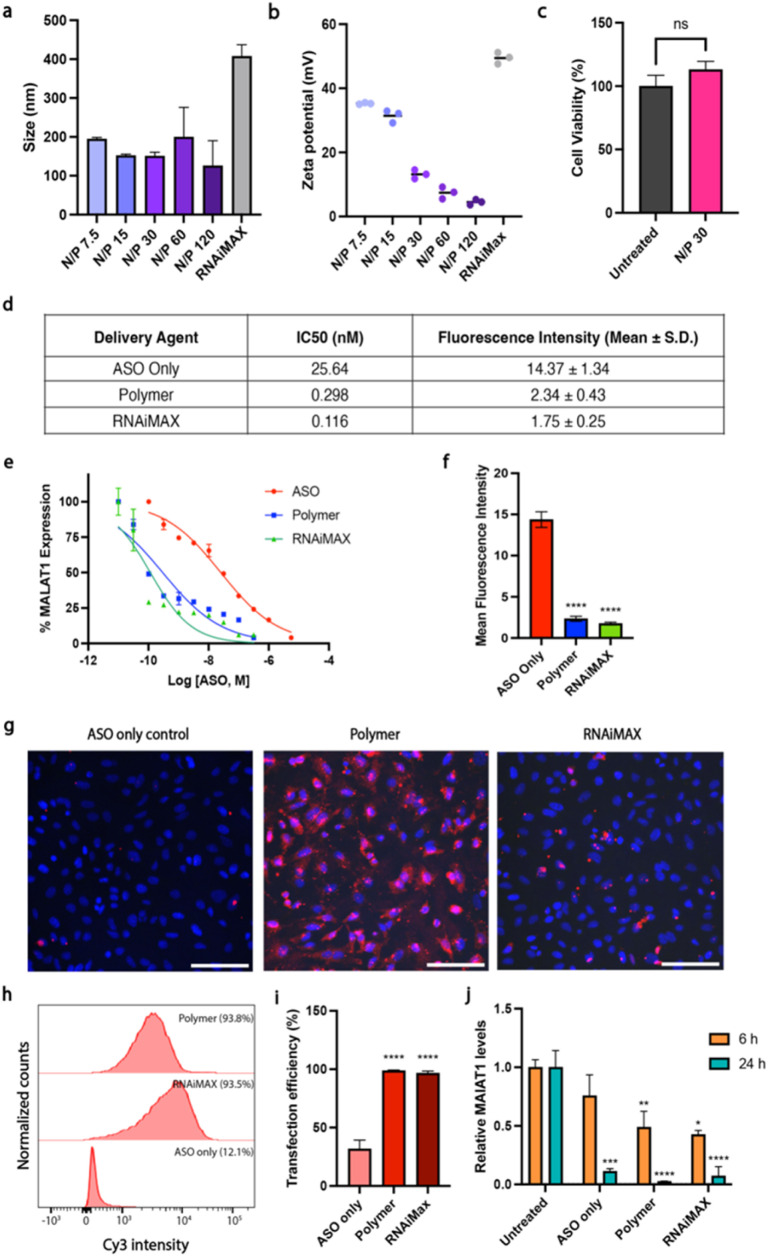
Polymer–ASO complexes enhance ASO uptake and activities *in vitro*. (a) Size of polymer–ASO complexes at various amine-to-phosphorothioates (N/P) ratios. Data shown as mean ± S.E.M. (b) Surface charge (zeta potential) of polymer–ASO complexes. Data shown as mean ± S.E.M. (c) Cell viability of HeLa cells after 24 h incubation with polymer–ASO complexes. Data from three technical replicates are shown as mean ± S.E.M. (d–f) Effect of ASO on *MALAT1* transcript levels in HeLa cells. Cy3-ASO fluorescence intensity (mean ± S.D.) in HeLa cells after 24 h treatment with IC_50_ concentrations calculated for *MALAT1* knockdown. (e) Expression levels were measured after treating cells for 24 h with different ASO concentrations. Data was normalised to *MALAT1* levels in untreated HeLa cells. (f) Mean ASO fluorescence intensity ± S.E.M. normalised to untreated control. Statistical significance is shown relative to the ASO only control: ****p ≤ 0.0001. (g) Representative fluorescence images of HeLa cells after 6 h incubation with polymer–ASO (N/P 30) complexes with ASO fluorescence (Cy3-ASO, red). (h) Representative flow cytometry histograms for the images presented in (g). (i) Both polymer and RNAiMAX samples had >98% transfection efficiencies after 6 h incubation, with ASO fluorescence quantified *via* flow cytometry. Data presented as mean ± S.D. from five replicates across two biological replicates, with 10 000 single cells collected per sample. Statistical significance shown relative to the ASO only control: ****p ≤ 0.0001. (j) Polymer and RNAiMAX transfections show ∼50% knockdown after 6 h and 24 h. Fold change of *MALAT1* mRNA expression, normalised to untreated control. Mean ± S.D. shown. Statistical significance is shown relative to the respective untreated control. Statistical significance calculated using ΔΔ*C*_t_ values, relative to untreated sample. ****p ≤ 0.0001, ***p ≤ 0.001, **p ≤ 0.01, *p ≤ 0.05, ns: *p* > 0.05.

Here, we used polymer and lipid delivery agents to transport a model gapmer 16 bp ASO (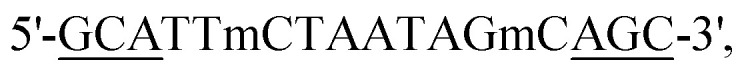 all phosphorothioate 3 + 10 + 3 gapmer, mC = 5-methylcytosine, underlined bases 2′-cEt) that induces RNase H-mediated degradation of the long non-coding RNA *MALAT1*. Polymer–ASO complexes were formed using our previously reported protocol, and were measured using dynamic light scattering (DLS), demonstrating that complexes were 100–200 nm in diameter at all tested N/P ratios, within the ideal range for endocytosis (Fig. 1a).

The surface charge (zeta potential) was also measured for the polymer–ASO complexes as a positive charge is required to facilitate endocytic uptake. The polymer–ASO complexes displayed overall positive surface charges, however the zeta potential declined with increasing N/P ratio (Fig. 1b), which may be an effect of increasing salt concentrations with increasing polymer, shielding the surface charge of the complexes in solution. An N/P 30 ratio was selected for the remaining cellular studies using this polymer delivery agent given that the size and charge of the complexes were within the optimal range and no toxicity was observed after 24 h (Fig. 1c). We then tested the activity and transfection efficiency of the polymer in HeLa human carcinoma cells using our model ASO targeting *MALAT1*. Lipofectamine RNAiMAX was used as a positive control given its known high transfection efficiency for siRNA and ASO gene knockdown studies. To characterise the relationship between the delivery systems and *MALAT1* knockdown efficiency, we conducted a dose–response study. We found that polymer and RNAiMAX-mediated ASO delivery had similar activities with IC_50_s (0.298 and 0.116 nM respectively) lower than the ASO only control (25.64 nM) (Fig. 1d and e). The IC_50_ values for polymer and RNAiMAX suggested enhanced delivery and uptake of ASOs into HeLa cells compared to free uptake, thus resulting in more efficient *MALAT1* knockdown. To investigate this further, we correlated ASO fluorescence intensity, measured *via* flow cytometry, to *MALAT1* transcript levels at the IC_50_ concentrations. While cells treated with ASO without a delivery agent displayed high ASO fluorescence, this did not translate to efficient *MALAT1* knockdown (Fig. 1d–f). In contrast, both polymer and RNAiMAX delivery agents exhibited lower ASO fluorescence intensities at the IC_50_ but had high knockdown efficiencies, indicating enhanced endosomal escape. This suggests that the polymer and RNAiMAX delivery display similar effectiveness in delivering ASOs into cells despite lower overall intracellular concentrations compared to free uptake. Since we have previously reported high ASO uptake at 5 μM without delivery agents,^17^ we used a lower concentration of 100 nM ASO for the cell studies to examine the effect of polymer and RNAiMAX delivery. Transfection efficiency was monitored by fluorescence microscopy and flow cytometry using a Cy3-tagged ASO (Fig. 1g–j). ASO fluorescence was visible in cells that had undergone transfection with either dendronised polymer or RNAiMAX (Fig. 1g). Cells that were incubated with ASOs only displayed a significantly lower uptake compared to those with polymer and RNAiMAX (*p* < 0.0001), both of which demonstrated almost 100% uptake efficiency (Fig. 1h and i).The activity of the ASOs was further assessed *via* quantitative reverse transcription–polymerase chain reaction (qRT-PCR). Polymer and RNAiMAX transfection with 100 nM ASO for 6 h resulted in *MALAT1* knockdown (Fig. 1j). After 6 h, both polymer and RNAiMAX significantly reduced *MALAT1* expression, while the ASO only control did not demonstrate a statistically significant effect of gene knockdown (*p* > 0.05). However, after 24 h incubation, all delivery mechanisms displayed significant *MALAT1* knockdown (*p* ≤ 0.001).

**Fig. 2:**
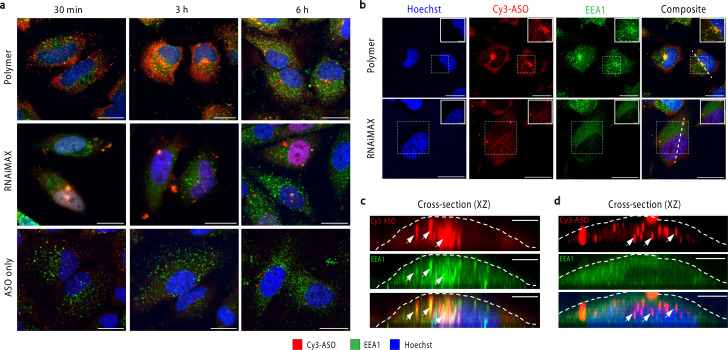
Visualisation of intracellular fate of polymer–ASO complex. (a) Representative confocal images of polymer and RNAiMAX transfection using 100 nM ASO after 30 min, 3 h, and 6 h incubations. ASO (red), EEA1 (green) and nuclei (blue), scale bar 20 μm. (b) After 3 h incubation of polymer–ASO complexes (top) and RNAiMAX–ASO complexes (bottom), the ASO (Cy3-ASO, red) colocalises with early endosomes (EEA1, green) at the perinuclear region, scale bar 20 μm. Boxed region indicates zoomed in image in top right corner, scale bar 5 μm. (c) Cross-section (*xz*) through the length of the polymer transfected cell as indicated by the white line in (b). White arrow indicates ASO and endosome colocalisation. The white dashed lines correspond with the cell boundary, scale bar 5 μm. (d) Cross-section (*xz*) through the length of the RNAiMAX transfected cell as indicated by the white line in (b). White arrows indicate ASO foci in the nucleus. The white dashed lines correspond with the cell boundary, scale bar 5 μm.

To investigate the intracellular trafficking of polymer and RNAiMAX transfections, we conducted a time-course experiment by collecting samples after 30 min, 3 h and 6 h incubations with HeLa cells (Fig. 2a). Early endosome antigen 1 (EEA1) and Rab7 were used to monitor ASO localisation with respect to early and late endosomes by confocal microscopy. Polymer–ASO complexes were observed to colocalise with early endosomes marked with EEA1, travelling from the periphery of cells towards the nucleus after 30 min incubation. It has been previously shown that cells with high uptake have ASO accumulation at the perinuclear region.^18^ Consistent with this, we observed that cells transfected with the polymer displayed ASO fluorescence within the cytosol and at the perinuclear region after 3 h (Fig. 2b and c). Diffusely distributed ASOs were also visible in both cytosol and nucleus in cells exposed to polymer–ASO complexes for 30 min, 3 h, and 6 h. After 24 h, ASO fluorescence accumulated in endolysosomal compartments for both polymer and RNAiMAX (ESI Fig. S1†). However, ASO fluorescence did not significantly colocalise with Rab7, a marker for late endosomes (ESI Fig. S2†). In contrast, RNAiMAX-facilitated delivery did not display colocalisation with either endosome marker, but ASO was detected within nuclei after only 3 h incubation (Fig. 2b and d). This is consistent with the delivery mechanism of RNAiMAX, whereby lipoplexes bypass endosomal encapsulation and release ASOs directly into the cytosol whence they are shuttled to different organelles. Here, ASO fluorescence was observed as bright, spherical foci, indicative of the formation of nuclear phosphorothioate bodies (PS-bodies).^19^ PS-bodies are accumulations of phosphorothioate ASOs (PS-ASOs) and TCP-1β protein that form irrespective of sequence and ASO activity.^20,21^ Of note, the segregation of ASOs in these PS-bodies does not appear to alter the silencing activities of ASOs.^22^

**Fig. 3:**
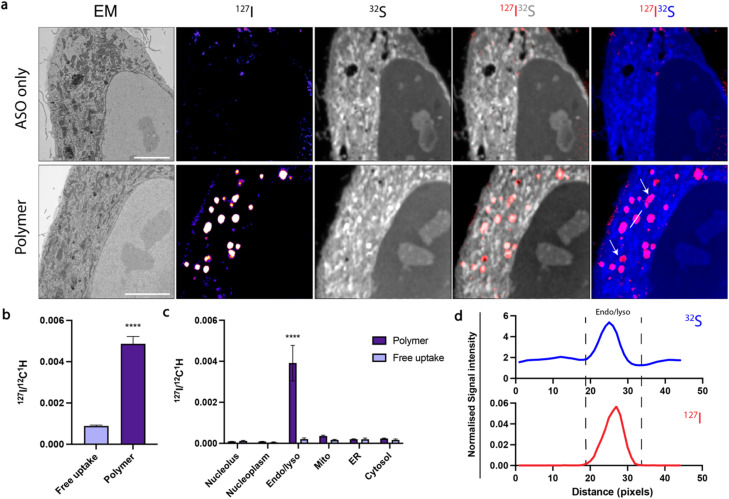
CEIM analysis demonstrates the polymer-facilitated increased cellular uptake of ASOs in endolysosomal compartments. (a) Scanning electron microscopy (SEM) and NanoSIMS images show that the polymer increases intracellular ASO signal. SEM image; iodine labelled ASO (^127^I NanoSIMS image); composite ^127^I (red) and ^32^S (grey); composite ^127^I (red) and ^32^S (blue). White arrows indicate ASO accumulation in endosomes. Scale bars 5 μm. (b) Comparison of the total ^127^I signal (ASO) per cell between free uptake and polymer delivery systems. Data presented as mean ± S.E.M. Number of cells counted *n* = 12–14. Statistical significance; ****p < 0.0001. (c) Quantification of ^127^I signal (ASO) normalised to ^12^C^1^H in subcellular organelles in HeLa cells. Data presented as mean ± S.E.M. *n* = 12–14. (d) ASO signal is colocalised to endolysosomal compartments (“Endo/lyso”) in polymer transfected cells. Area measured is indicated by white line in polymer ^127^I^32^S image in (a); 1 px = 78.125 nm.

Confocal microscopy suggested that polymer and RNAiMAX delivery agents may have different intracellular trafficking mechanisms, which could result in differing final organelle distributions. We therefore turned to the CEIM workflow to further investigate the intracellular delivery of ASOs by polymer and RNAiMAX. We first showed that ASOs delivered by RNAiMAX primarily accumulated in the nucleoplasm with accumulations in PS-bodies (ESI Fig. S3†), which is consistent with our previous report.^17^ Next, we aimed to profile the organelle-specific distributions of ASOs mediated by polymer. For NanoSIMS experiments, the ASOs were labelled with iodine (^127^I) which was incorporated as 5-iododeoxyuridine (IdU), which were substituted for two dT nucleotides in the ASO. We have previously confirmed that halo-dT modifications to the ASO do not affect *MALAT1* knockdown efficacy.^17^ The use of our polymer delivery agent dramatically increased the internalisation of ASO after 6 h incubation in HeLa cells, as compared with the free uptake group without delivery agent (Fig. 3a and b). This is consistent with the flow cytometry quantification, confocal imaging, as well as the qPCR assays (Fig. 1). Cells transfected with ASOs using polymer delivery agent showed a significant accumulation of ASO signal within endosomal compartments compared to all other organelles (Fig. 3c). The identification of endolysosomal compartments by electron microscopy ultrastructure were supported by our observation that ASO signals overlapped with regions enriched in sulfur, characteristic of endolysosome compartments (Fig. 3d).

In order to perform gene silencing activities, ASOs must separate from the polymer to reach the cytosol and nucleus. However, it is currently unclear at what stage and how this intracellular trafficking process occurs. To address these challenges, we additionally modified our polymer, incorporating bromine through reaction of 10% dendron primary amines with 4-bromobenzoic acid to enable NanoSIMS detection (Br-polymer) (ESI Fig. S5 and S6†). While bromine (Br) labelling increased the size of polymer–ASO complexes, the charge at N/P 30 was consistent with the unmodified polymer. Following delivery of polymer–ASO complexes, the polymer was distributed both inside and outside endolysosomal compartments (ESI Fig. S4e†). This observation could be expected, since at N/P 30, there is likely to be a substantial excess of free polymer in addition to the polymer–ASO complexes.

**Fig. 4:**
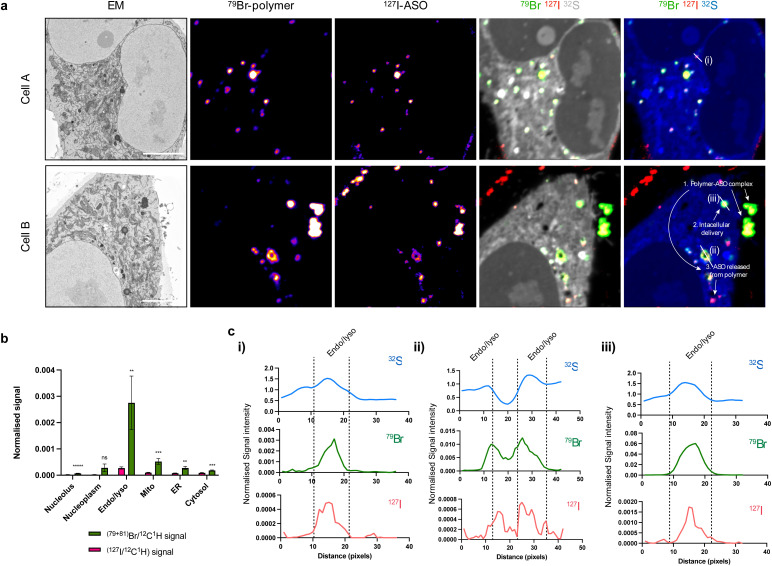
Examining polymer and ASO separation within cells. (a) SEM and NanoSIMS images showing the release of ASO from polymer after 6 h incubation in the cytoplasm of HeLa cells. SEM image; Br-polymer (^79^Br NanoSIMS image), I-ASO (^127^I NanoSIMS image); composite ^79^Br (green), ^127^I (red) and ^32^S (grey); composite ^79^Br (green), ^127^I (red) and ^32^S (blue). Scale bars 5 μm. (b) Polymer (^79^Br/^12^C^1^H) and ASO (^127^I/^12^C^1^H) signals are strongest in endosome/lysosome (“Endo/lyso”) compartments. Data presented as mean ± S.E.M (*n* = 12). Statistical significance calculated using two-way ANOVA multiple comparisons test: **p < 0.01, ***p < 0.001, ****p < 0.0001, ns = not significant. (c) Distribution of ^32^S, ^79^Br (polymer), and ^127^I (ASO) signals over areas indicated by the white lines in (a), (i–iii) respectively; 1 px = 78.125 nm. ^32^S, ^79^Br, and ^127^I signals are normalised to ^12^C^1^H.

Overlap of polymer and ASO signals, indicative of the presence of a polymer–ASO complex, were observed both inside and outside cells (Fig. 4a). Again, a significant cellular uptake of ASOs facilitated by polymer was observed. Not surprisingly, both polymer and ASO signals were enriched in endolysosomal compartments compared to other organelles (Fig. 4b). Interestingly, intensities of polymer signals and ASO signals within each endolysosome vary, suggesting that polymer and ASO separation may occur within these compartments.

Further investigation within endolysosomes regions indicated that ASO begins to separate from its polymer carrier (Fig. 4c). Plotting polymer and ASO signal with respect to high sulfur intensities (indicative of endosomes and/or lysosomes) showed an offset of Br (polymer) and I (ASO) peaks. This suggests that the polymer and ASO are separated within these compartments and free ASO is being released. We have previously shown that with decreasing pH, polymer binding is enhanced through an increase in primary and/or tertiary amine ionisation resulting in an increased positive surface charge.^23^ Early endosomes have a pH ∼ 6.5, suggesting that binding should be strengthened under such conditions and that there must be other factors involved in facilitating polymer and ASO dissociation. In addition to accumulation in endosomal compartments, we also observed Br and I signal in clusters with low S intensity regions (Fig. 4c(ii and iii)). We suspect that these may be cytoplasmic processing bodies (P-bodies) or stress granules which have the ability to form structures with PS-ASOs.^24,25^

## Conclusions

ASOs offer an approach for suppressing the expression of specific genes, but require delivery tools to cross the cell membrane and enter the cytoplasm, from where they can access complementary RNA targets. We demonstrate that while polymer and lipid-based delivery agents both enhance ASO uptake, the choice of delivery agent results in different intracellular trafficking routes. We also report visualisation of drug carriers and therapeutics in the same cells and, for the first time, the separation of polymer and ASO within cells at the nanoscale. Our findings suggest that this separation occurs within endosomal compartments, with free ASO released into the cytosol. This information enables a better understanding of the intracellular trafficking pathways of nucleic acid therapeutics and may be exploited for therapeutic delivery to enhance the effectiveness of nucleic acid therapeutics in future.

## Experimental

### Preparation and characterisation of polymer–ASO complexes

The dendritic polymer was synthesised as per Kretzmann *et al.*^11^ using a 25 mol% GMA backbone and generation 5.0 poly(amido amine) dendrons. Polymer–ASO complexes were assessed *via* dynamic light scattering and zeta potential measurements. Polymer solutions were mixed with 1 μg ASO at multiple amine-to-phosphorothioate (N/P) ratios and incubated at 25 °C for 30 min. Solutions were diluted to 900 μL and measurements were taken in triplicate. Reported values are the mean ± SD for average peak size and zeta potential.

### Bromine-modified polymer synthesis

4-Bromobenzoic acid (1.00 g, 4.97 mmol) was taken up in dry DCM (15 mL), cooled to 0 °C and DMF (1 drop) added, followed by dropwise addition of oxalyl chloride (0.56 mL, 6.53 mmol). The reaction was allowed to warm to room temperature and left stirring overnight. The reaction mixture was concentrated under reduced pressure to afford 4-bromobenzoyl chloride in quantitative yield. Next, generation 5 PAMAM dendron functionalised polymer (20.0 mg, ≈85.1 μmol NH_2_) was taken up in dry DMF (2 mL), then 4-bromobenzoyl chloride (1.86 mg, 8.5 μmol) added and the reaction left stirring overnight. The reaction was quenched with DI water (2 mL), transferred to dialysis tubing (MWCO 10 kDa), and dialysed against DI water (4 × 5 L) over 16 h. The product was concentrated by lyophilisation to afford brominated dendronised polymer (17.2 mg) with 10% of amines substituted as determined by NMR.

### Cell culture

HeLa cells (ATCC) were cultured in Minimum Essential Medium α (Gibco cat# 12571071) supplemented with 10% FBS at 37 °C and 5% CO_2_. HeLa cells were maintained at 37 °C in a humidified incubator with 5% CO_2_ and passaged at 70–80% confluency using 1× TrypLE Express.

### Transfection

HeLa cells were seeded at 150 000 cells per mL in a 24-well plate and left overnight at 37 °C and 5% CO_2_. Polymer (2 mg mL^−1^ stock solution) was diluted in 35 μL Opti-MEM (Gibco #31985070). ASO was diluted to 23 pmol per well in 35 μL Opti-MEM. Polymer and ASO solutions were mixed gently and incubated for 25 min at room temperature. For positive control, RNAiMAX was diluted in 35 μL Opti-MEM as per manufacturer's protocol and mixed with an equal volume of 23 pmol ASO. ASO only samples were incubated with 100 nM ASO in 215 μL Opti-MEM and untreated control samples were incubated in 215 μL Opti-MEM. Cells were washed with 1× PBS once to remove serum and media was replaced with 150 μL Opti-MEM. 65 μL of polymer–ASO complexes were added to cells (100 nM ASO final concentration) and incubated for 4 h at 37 °C. For all samples, after 4 h 250 μL media was added to each well and cells were incubated for a further 2 h. After the full 6 h incubation, transfection efficiency was visualised with an epifluorescence microscope. For quantification *via* flow cytometry, cells were incubated with 250 μL 1× TrypLE for 10 min and collected with 250 μL 2× FACS buffer (6% v/v FBS, 2 mM EDTA in 1× PBS).

### Flow cytometry

Cells were harvested using 250 μL 1× TrypLE for 10 min and collected with 250 μL 2× FACS buffer. Typically, 10 000 single cells were gated on FSC-A *vs.* SSC-A and then SSC-H *vs.* SSC-A were counted by flow cytometry (BD Canto II). For ASO uptake studies, single cells were measured for Cy3-ASO fluorescence using 488 nm excitation and 585/42 nm emission filter. For fluorescence intensity studies using IC_50_ concentrations, cells were processed using a two-camera Amnis ImageStreamX mark II flow cytometer (ISXmkII) with INSPIRE v6.1 acquisition software (Cytek Biosciences, USA). Cy3-ASO fluorescence was measured using 561 nm laser and emission captured in the range between 560–595 nm.

### RNA extraction, cDNA conversion and qRT-PCR

Cells were treated with outlined in the transfection protocol. After 6 h incubation, cells were washed twice with 1× PBS. The Qiagen RNeasy Mini Kit (#74104) was used for cell lysis and RNA extraction. For cell lysis 350 μL of the Qiagen RLT lysis buffer were added to each well, pipetted to lyse cells, and transferred to 1.5 mL tubes. Lysates were stored at −80 °C or continued straight to RNA extraction. DNA was eliminated using Qiagen's RNAse-Free DNase kit (#79254). RNA concentration and purity was assessed using a NanoDrop Microvolume UV-vis Spectrophotometer. RNA was converted to cDNA using Qiagen's QuantiTect Reverse Transcriptase Kit, according to the manufacturer's instructions (#205313). qRT-PCR was conducted using Qiagen's SYBR Green Master Mix (#208056) with *GAPDH* and *MALAT1* gene-specific primers. Each condition was plated in technical triplicates. *MALAT1* Cp values were normalised against *GAPDH*, and samples were standardised to the untreated (no ASO) control group. Statistical significance was calculated with *t*-test; *: *p* ≤ 0.05, ***: *p* ≤ 0.001, ns: *p* > 0.05.

### IC_50_ determination

HeLa cells were seeded and treated with ASOs *via* the three methods outlined in the transfection section; free uptake, polymer-mediated, and RNAiMax-mediated. A 10-point 0.5-log dilution series of ASOs was prepared in Opti-MEM. Treated cells were incubated for 24 h under standard conditions (37 °C, 5% CO_2_). Following incubation, cells were harvested, lysed and processed according to the RNA extraction, cDNA conversion and qRT-PCR protocols. *MALAT1* levels were normalized to control using GAPDH and B2M as housekeeping genes. IC_50_ values were calculated using GraphPad Prism v10. Relative expression levels of *MALAT1* were plotted against the ASO concentrations, which were log-transformed. Nonlinear regression analysis was performed using the “log(inhibitor) *vs.* response – variable slope (four parameters)” model. The IC_50_, representing the concentration of ASO required to achieve 50% knockdown of *MALAT1*, was calculated for each treatment group (free uptake, polymer, and RNAiMAX). Data are presented as mean ± standard deviation from technical replicates.

### Endosomal immunofluorescence

HeLa cells were plated on sterile glass coverslips at 160 000 cells per well in a 24-well plate and left overnight at 37 °C and 5% CO_2_. Transfections were conducted as described in the transfection protocol section, with multiple incubation times (30 min, 3 h, 6 h, 24 h). At the end of the incubation, cells were washed briefly twice with 1× PBS. Cells were fixed in 4% w/v PFA in 1× PBS for 15 min at 37 °C. Cells were washed with 1× PBS twice, 5 min each and permeabilised in 1% saponin in 1× PBS for 5 min. Next, cells were washed with 1× PBS 2 × 10 s each prior to blocking in 3% BSA in 1× PBS for 30 min. Primary antibodies were diluted in 0.1% saponin, 0.2% BSA in 1× PBS for 2 h. Mouse anti-EEA-1 (BD Biosciences, #610457) was diluted 1 : 200 and mouse anti-Rab7 (Cell Signaling (E907E), #95746) was diluted at 1 : 100. After antibody incubation, cells were washed 0.2% BSA in 1× PBS 3 × 5 min. Secondary antibody donkey anti-mouse IgG (H + L) Alexa Fluor 488 (Invitrogen, #A21202) (1 : 500) were diluted in 0.1% w/v saponin, 0.2% w/v BSA in 1× PBS for 45 min. Cells were washed 0.2% w/v BSA in 1× PBS 3 × 5 min. Hoechst 34580 was diluted in 1× PBS (1 μg mL^−1^) and added to cells for 10 min. Finally, coverslips were briefly washed 3 times with 1× PBS and mounted onto microscope slides with Fluoromount G. Samples were imaged with Nikon A1R with PicoQuant FCS/FLIM in Ti-E microscope (CMCA, UWA) using a 100× oil immersion objective and analysed using ImageJ.

### SEM and NanoSIMS sample preparation

For SEM and NanoSIMS cells were cultured on coverslips and processed as outlined in He *et al.*, (2021).^17^ Briefly, cells were transfected with I-ASO and washed with warm fixative solution (2.5% glutaraldehyde in 0.1 M sodium cacodylate). Cells were fixed in fresh fixative solution for 10 min at room temperature, followed by 2 h on ice. Cells were washed with cold 0.1 M sodium cacodylate 5 times (3 min each) and post-fixed with 2% osmium tetroxide in 0.1 M sodium cacodylate for 1 h on ice. Next, cells were washed with cold distilled water 5 times (3 min each) and incubated in 1% thiocarbohydrazide solution for 20 min at room temperature. The coverslips were transferred to clean wells and washed with distilled water at room temperature (5 times, 3 min each). The coverslips were then incubated in 2% osmium tetroxide at room temperature for 30 min, followed by washing with cold distilled water (5 times, 3 min each). Next, the coverslips were incubated with 2% aqueous uranyl acetate overnight at 4 °C. The following day, the cells were washed (5 times, 3 min each) with cold distilled water and dehydrated with increasing amounts of ethanol (30%, 50%, 70%, 85%, 95%, 100%) for 2 min each, followed by two 2 min incubations with 100% ethanol. The samples were then infiltrated with Embed812 resin (Electron Microscopy Sciences) by incubating samples in 50% resin (diluted in anhydrous acetone) for 1 h, 66% resin overnight, and finally 100% resin for 2 h. Coverslips were inverted onto individual BEEM capsules (Electron Microscopy Sciences) which had been filled with resin. Capsules with coverslips were polymerised in a vacuum oven for 48 h at 65 °C. To prepare samples for sectioning, the BEEM capsules were cut away from the polymerised resin with a razor blade. The coverslip was removed from the resin block and the block was trimmed; 500 nm sections were cut with a Leica UC6 ultramicrotome with a Diatome diamond knife.

### SEM and NanoSIMS imaging

The images of cell ultrastructure were collected under an FEI Verios scanning electron microscope (SEM) (Thermo Fisher Scientific) using backscattered electrons (BSEs). To map the cellular ultrastructure with the chemical ions, the sections were coated with 5 nm gold and performed NanoSIMS (NanoSIMS 50 or NanoSIMS 50L, CAMECA, France) analysis in the same areas of interest. Primary ions using ^133^Cs^+^ beam (A 16 keV) were used to bombard the sample surface, and secondary ions or ion clusters (^12^C^1^H, ^12^C^14^N^−^, ^32^S^−^, ^79^Br^−^, ^127^I^−^, *etc.*) were captured by the multicollection ion detectors to create the ion images. Firstly, ^133^Cs^+^ implantation was performed using the following settings to remove the gold coating and ensure the steady release of secondary ions: a dose of ∼1 × 10^17^ ions per cm^2^, primary ion beam current of ∼1 nA using primary aperture *D*1 = 1. Regions of 20 × 20 μm were imaged with an 8 pA beam current (primary aperture *D*1 = 2) and a dwell time of 3.0 ms per px to obtain 256 × 256 px images. The image preparation and quantification were performed in Open-MIMIS plugin in ImageJ. Ion images of ^12^C^14^N^−^ and ^32^S^−^ were used to display the cell morphology and images of ^79^Br^−^ and ^127^I^−^ were used to demonstrate the distributions of polymer or ASOs as indicated in the figures.

## Data availability

The data that support the findings of this study are available on Open Science Framework (OSF) at DOI 10.17605/OSF.IO/NMXBY, DOI 10.17605/OSF.IO/6FJBA, and DOI 10.17605/OSF.IO/P64NB, project reference ‘ASO trafficking (1–3)’.

## Author contributions

K. S. I., C. W. E., and N. M. S. conceived and supervised the project. J. J. K., K. S. I., C. W. E., K. C. and H. J. designed experiments. K. S. I., H. J., S. G. Y., M. Ni and C. W. E. secured resources and funding. J. J. K., C. W. E., U. B., R. A., Q. L., H. YL. H., N. J. P., K. C., M. No. and M. Ni. performed experiments. J. J. K., C. W. E., K. C., K. S. I., S. G. Y. and H. J. analyzed the data. C. W. E., J. J. K., K. S. I. and H. J. wrote the manuscript. All authors contributed to editing the manuscript.

## Conflicts of interest

There are no conflicts to declare.

## Supplementary Material

SC-OLF-D3SC06773D-s001
