# Supplementary material for: High-resolution visualisation of antisense oligonucleotide release from polymers in cells
Source: Chem Sci. 2024 Aug 28;15(38):15690–7. doi: 10.1039/d3sc06773d (PMC11378015; doi:10.1039/d3sc06773d)
Supplement: SC-015-D3SC06773D-s001 [file SC-015-D3SC06773D-s001.pdf]

Supplementary Information (SI) for Chemical Science.
This journal is © The Royal Society of Chemistry 2024

Supporting Information

High-Resolution Visualisation of Antisense Oligonucleotide Release from Polymers in Cells

Jessica J. King[‡a,b], Kai Chen[‡a,b], Cameron W. Evans[a,b], Marck Norret[a,b], Ruba Almasri[a,b,c] Nathan J. Pavlos[d], Henry Y.L. Hui[e], Qiongxiang Lin[a,b], Uditi Bhatt[a,b], Stephen G. Young[f], Nicole M. Smith[a,b], Mehran Nikan[g], Clive A. Prestidge[c], Haibo Jiang[*a,b,h], K. Swaminathan Iyer[*a,b]

[a] School of Molecular Sciences, The University of Western Australia, Perth WA 6009, Australia.

[b] ARC Training Centre for Next-Generation in Biomedical Analysis, The University of Western Australia, Perth WA 6009, Australia

[c] UniSA Clinical & Health Sciences, University of South Australia, Adelaide SA, Australia

[d] School of Biomedical Sciences, The University of Western Australia, Perth WA 6009, Australia.

[e] Translational Cancer Pathology Laboratory, School of Biomedical Sciences, The University of Western Australia, Perth WA 6009, Australia

[f] Department of Medicine, University of California, Los Angeles, CA 90095, USA.

[g] Ionis Pharmaceuticals, Inc., Carlsbad, CA 92010, USA.

[h] Department of Chemistry, Faculty of Science, University of Hong Kong, Pok Fu Lam, Hong Kong.

Contents

Supplementary Figure S1. Diffuse distribution of ASO after 24 h results in efficient knockdown activity. Representative confocal images of Cy3-ASO (100 nM) delivery into cells after 24 h incubation. ASO (Cy3, red), EEA1 (Alexa Fluor 488, green) and nuclei (Hoechst 34580, blue). Scale bar 10 µm. S3

Supplementary Figure S2. Representative confocal images of Cy3-labelled *MALAT1* ASO (100 nM) delivery into cells after 30 min, 3 h, 6 h, and 24 h incubation. ASO-Cy3 (red), Rab7 (green) and nuclei (blue). Scale bar 20 µm. S4

Supplementary Figure S3. RNAiMAX delivers ASO into nucleus of HeLa cells. **(a)** Scanning electron microscopy (SEM) and NanoSIMS images showing the delivery of ASO into the nucleus of HeLa cells by RNAiMAX. SEM image, bromine labelled ASO (^{79Br} NanoSIMS image), composite ^{79Br} (red), ^{12C14N} (grey). **(b)** Quantification of ASO (^{79Br} signal) in subcellular compartments. S5

Supplementary Figure S4. Bromine labelled polymer facilitated polymer tracking via NanoSIMS. **(a)** Bromine labelling of generation 5 (G5) dendronised polymer. **(b)** Size of polyplex complexes at various N/P ratios. Data shown as mean ± S.E.M. **(c)** Surface charge (zeta potential) of polyplexes at different N/P ratios. Data shown as mean ± S.E.M. **(d)** Quantification of bromine (*i.e.,* polymer) signal, collected from 10 cells. **(e)** Representative images of the localisation of bromine labelled polymer (unbound to ASO) in cells. SEM image, bromine labelled polymer (^{79Br} NanoSIMS image), composite ^{79Br} (red), ^{32S} (grey), composite ^{79Br} (green), ^{32S} (blue). S6

Supplementary Figure S5. ^{1H} NMR spectra (500 MHz, MeOD, 25 °C) of G5 PAMAM dendronised polymer (top), and bromobenzyl modified polymer used for NanoSIMS (bottom). The appearance of signals at δH 7.73–7.61 ppm (blue shaded region) confirms polymer labelling with Br. S7

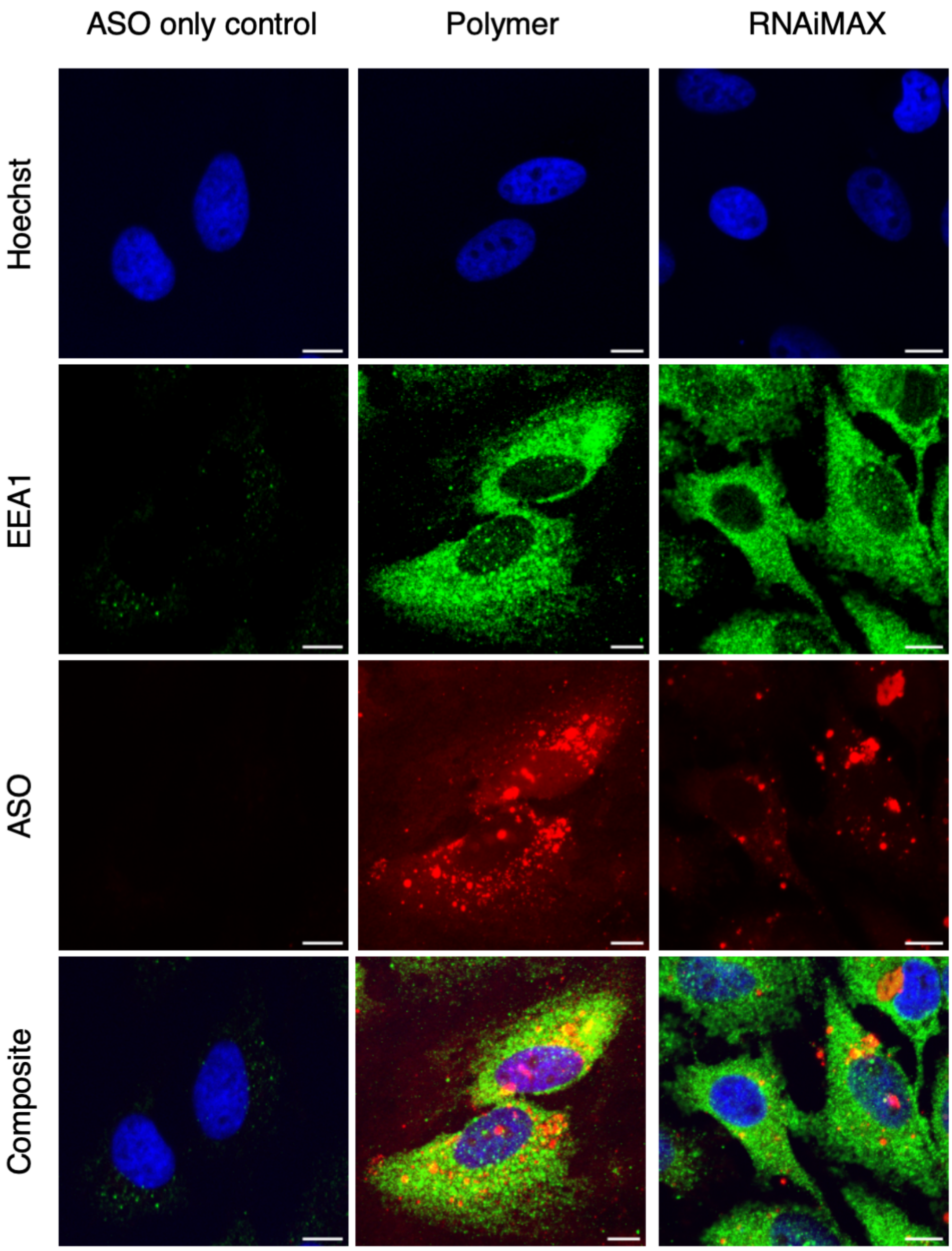

Supplementary Figure S1. Diffuse distribution of ASO after 24 h results in efficient knockdown activity. Representative confocal images of Cy3-ASO (100 nM) delivery into cells after 24 h incubation. ASO (Cy3, red), EEA1 (Alexa Fluor 488, green) and nuclei (Hoechst 34580, blue). Scale bar 10 µm.

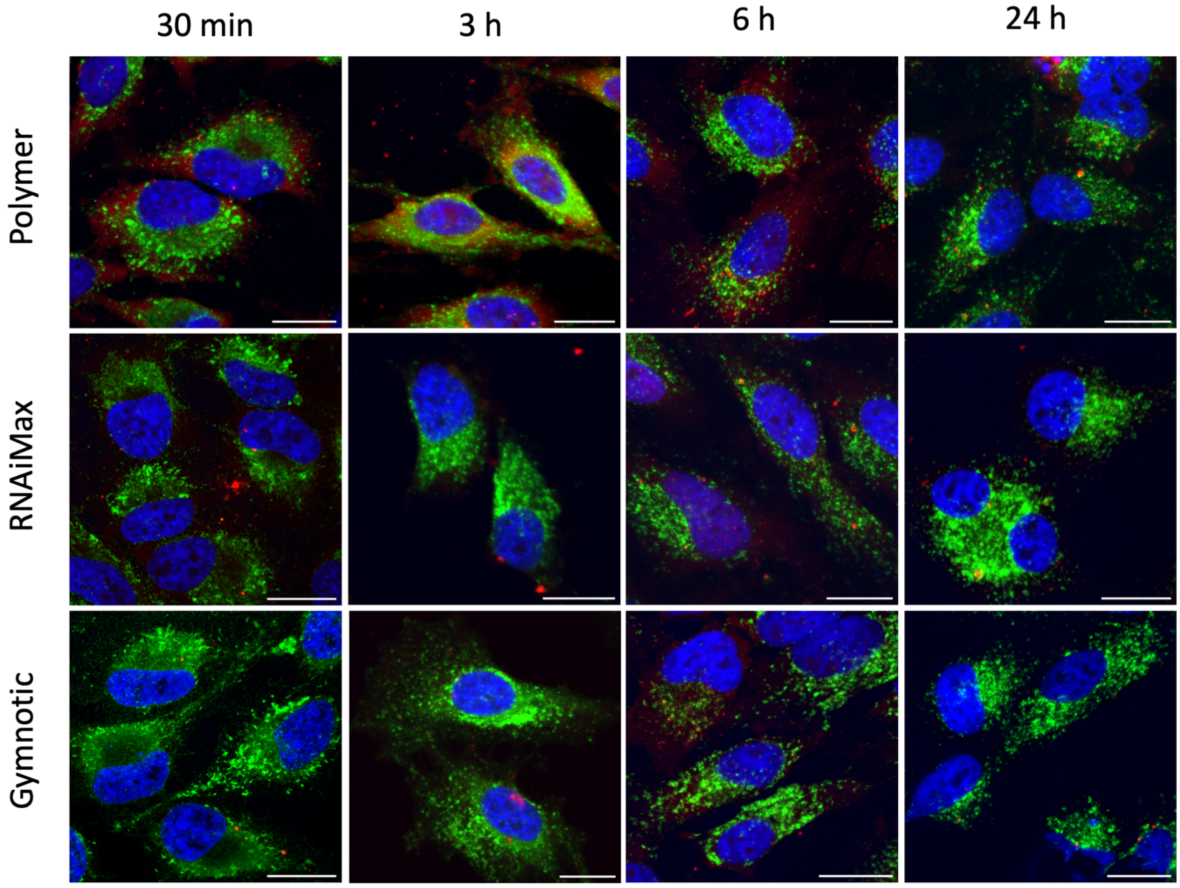

Supplementary Figure S2. Representative confocal images of Cy3-labelled *MALAT1* ASO (100 nM) delivery into cells after 30 min, 3 h, 6 h, and 24 h incubation. ASO-Cy3 (red), Rab7 (green) and nuclei (blue). Scale bar 20 µm.

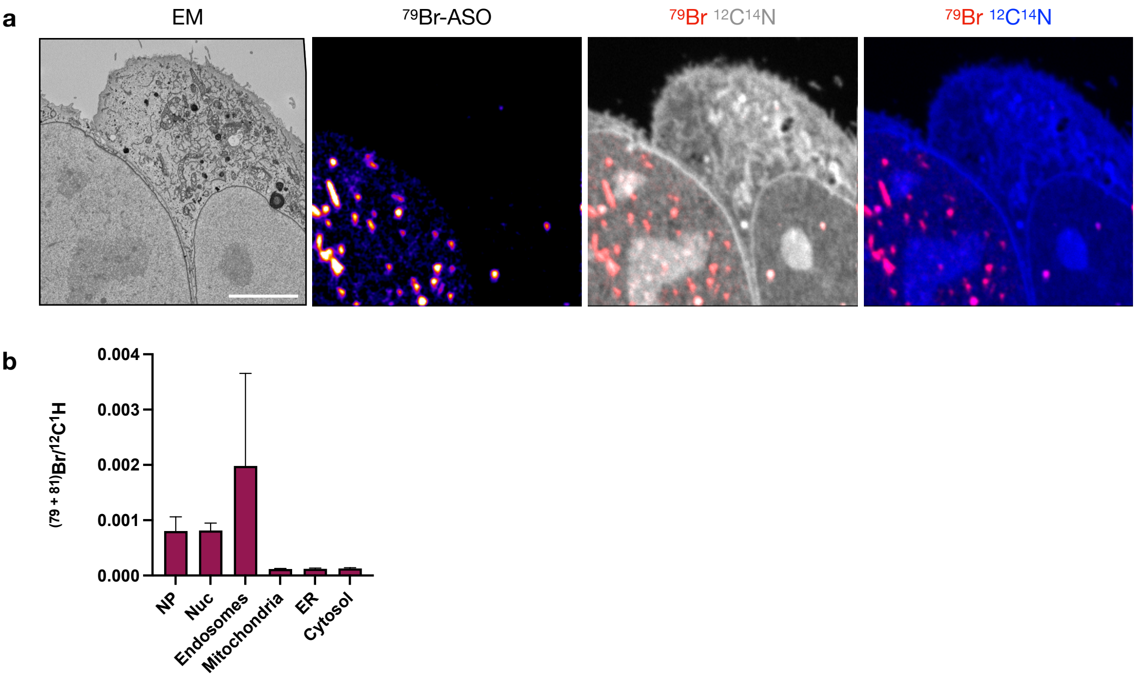

Supplementary Figure S3. RNAiMAX delivers ASO into nucleus of HeLa cells. **(a)** Scanning electron microscopy (SEM) and NanoSIMS images showing the delivery of ASO into the nucleus of HeLa cells by RNAiMAX. SEM image, bromine labelled ASO (^{79}Br NanoSIMS image), composite ^{79}Br (red), ^{12}C^{14}N (grey). **(b)** Quantification of ASO (^{79}Br signal) in subcellular compartments.

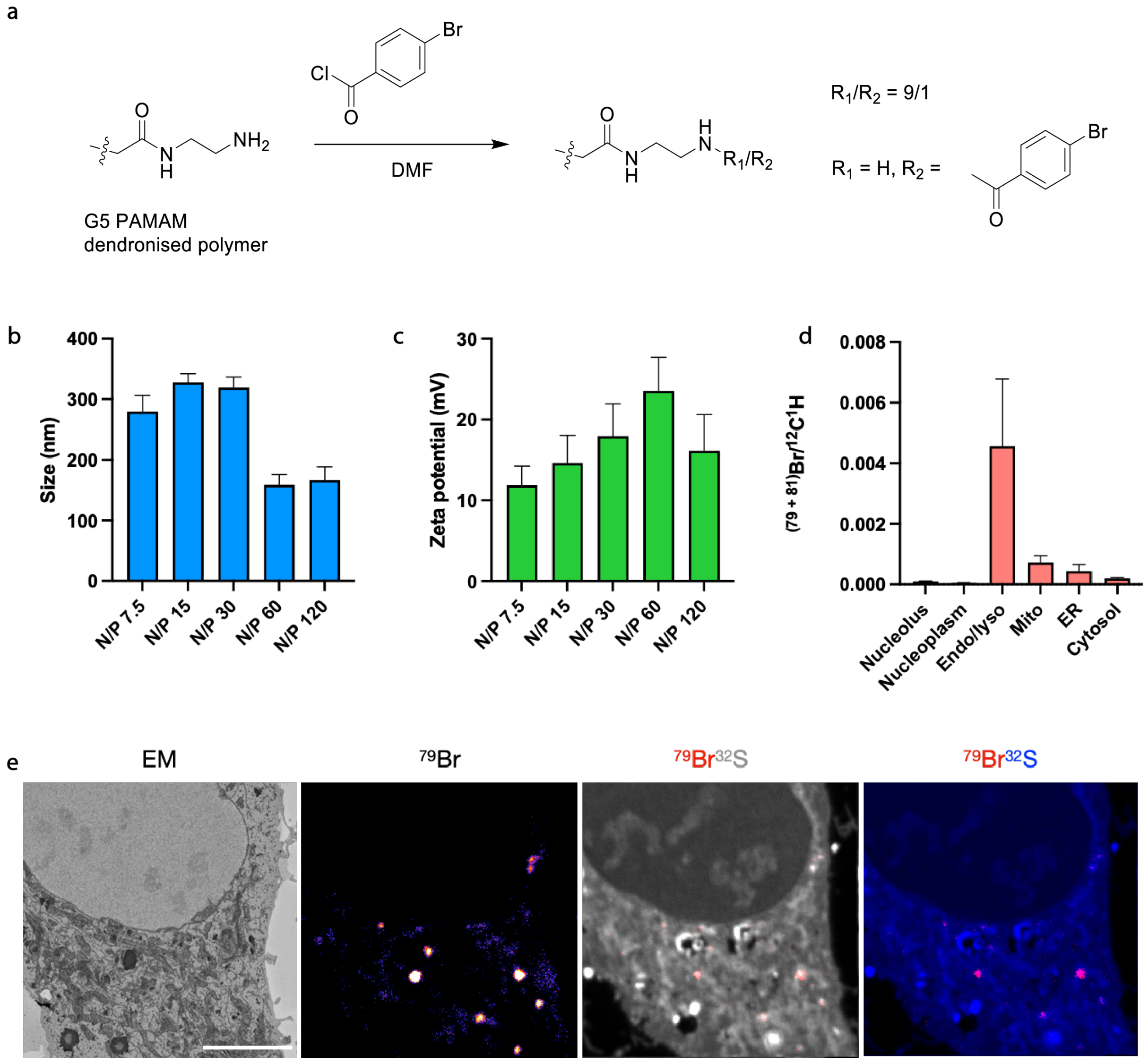

Supplementary Figure S4. Bromine labelled polymer facilitated polymer tracking via NanoSIMS. **(a)** Bromine labelling of generation 5 (G5) dendronised polymer. **(b)** Size of polyplex complexes at various N/P ratios. Data shown as mean ± S.E.M. **(c)** Surface charge (zeta potential) of polyplexes at different N/P ratios. Data shown as mean ± S.E.M. **(d)** Quantification of bromine (*i.e.,* polymer) signal, collected from 10 cells. **(e)** Representative images of the localisation of bromine labelled polymer (unbound to ASO) in cells. SEM image, bromine labelled polymer (^{79}Br NanoSIMS image), composite ^{79}Br (red), ^{32}S (grey), composite ^{79}Br (green), ^{32}S (blue).

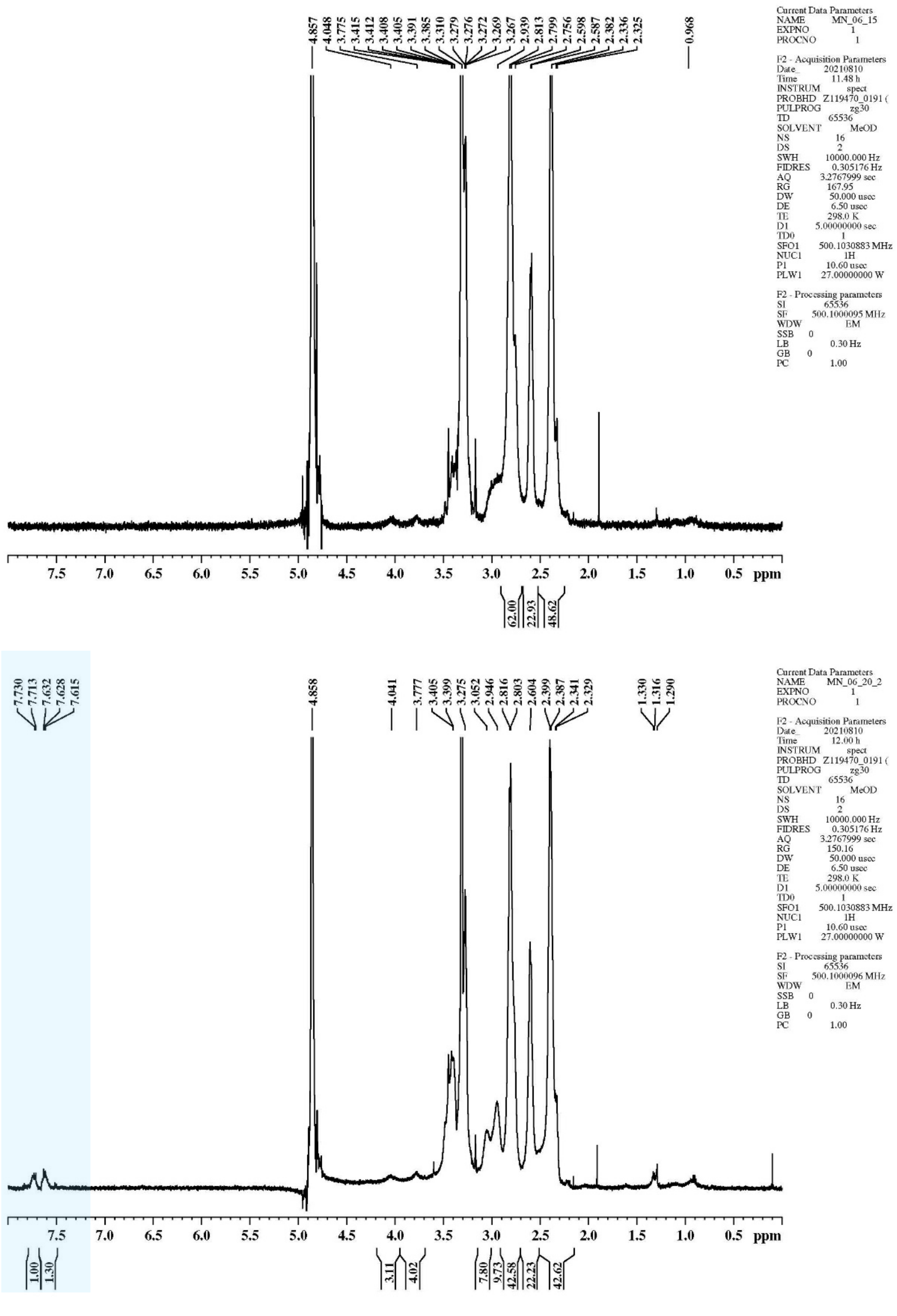

Supplementary Figure S5. ^{1}H NMR spectra (500 MHz, MeOD, 25 °C) of G5 PAMAM dendronised polymer (top), and bromobenzyl modified polymer used for NanoSIMS (bottom). The appearance of signals at δ_H 7.73–7.61 ppm (blue shaded region) confirms polymer labelling with Br.
